# Year‐Round Quantification, Structure and Dynamics of Epibacterial Communities From Diverse Macroalgae Reveal a Persistent Core Microbiota and Strong Host Specificities

**DOI:** 10.1111/1758-2229.70077

**Published:** 2025-03-12

**Authors:** Maéva Brunet, Nolwen Le Duff, Tristan Barbeyron, François Thomas

**Affiliations:** ^1^ Sorbonne Université, CNRS, Integrative Biology of Marine Models (LBI2M) Station Biologique de Roscoff (SBR) Roscoff France

**Keywords:** algae, bacteria, holobionts, microbe: higher organism interactions, microbial communities

## Abstract

Macroalgae‐bacteria interactions play pivotal ecological roles in coastal ecosystems. Previous characterisation of surface microbiota from various macroalgae evidenced fluctuations based on host tissues, physicochemical and environmental parameters. However, the dynamics and degree of similarity of epibacterial communities colonising phylogenetically distant algae from the same habitat are still elusive. We conducted a year‐long monthly epimicrobiota sampling on five algal species inhabiting an English Channel rocky shore: *Laminaria digitata* , *Ascophyllum nodosum* , *Fucus serratus* (brown algae), *Palmaria palmata* (red alga) and *Ulva* sp. (green alga). To go beyond relative compositional data and estimate absolute variations in taxa abundance, we combined qPCR measurements of 16S rRNA gene copies with amplicon metabarcoding. A core microbiome composed of 10 genera was consistently found year‐round on all algae. Notably, the abundant genus *Granulosicoccus* stood out for being the only one present in all samples and displayed an important microdiversity. Algal host emerged as the primary driver of epibacterial community composition, before seasonality, and bacterial taxa specifically associated with one or several algae were identified. Moreover, the impact of seasons on the epimicrobiota varied depending on algal tissues. Overall, this study provides an extensive characterisation of the microbiota of intertidal macroalgae and enhances our understanding of algal‐bacteria holobionts.

## Graphical abstract

We conducted a year‐long monthly epimicrobiota sampling on five algal species inhabiting an English Channel rocky shore, combining qPCR measurements of 16S rRNA gene copies with amplicon metabarcoding. Algal host emerged as the primary driver of epibacterial community composition, before seasonality. A core microbiome composed of 10 genera was consistently found year‐round on all algae.

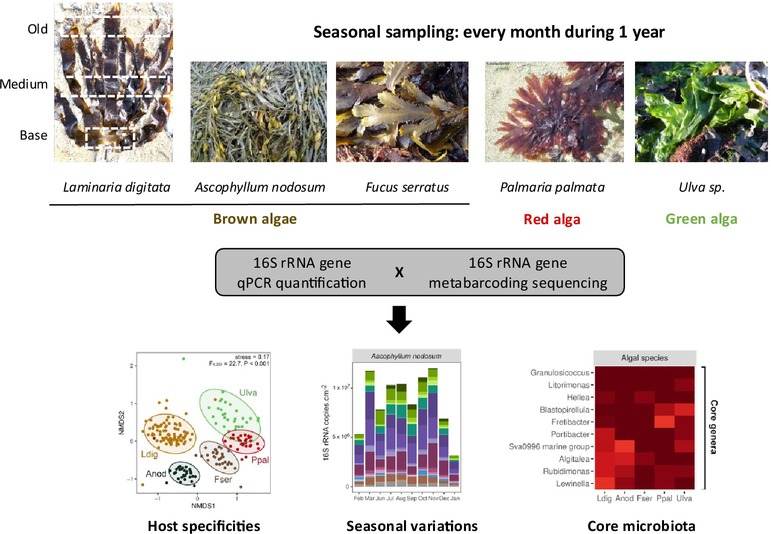

## 1 Introduction

Macroalgae play crucial roles in marine ecosystems, contributing significantly to primary production, nutrient cycling, and habitat provision. Their surface harbours a dense, complex and highly dynamic biofilm of bacteria, archaea, fungi and viruses. Bacteria dominate the microbiota in abundance and diversity, representing up to 10^8^ cells cm^−2^ (Egan et al. 2013; Martin et al. 2015), and form intricate interactions with macroalgae. These associations were reported to be either beneficial, detrimental or commensal (Burgunter‐Delamare et al. 2024) and are at the basis of various essential processes in coastal oceans, including roles in nutrient cycling (Brunet et al. 2022) and in shaping host morphology, reproduction and defence against pathogens (Wichard et al. 2015; Singh and Reddy 2016). The structure and composition of the microbiota of various brown (e.g., Bengtsson et al. 2012; Florez et al. 2019; Burgunter‐Delamare et al. 2023), red (Miranda et al. 2013; Zozaya‐Valdés et al. 2017) and green (Burke et al. 2011; Van der Loos et al. 2023) macroalgae have been largely investigated in the past decades using culture‐dependent and ‐independent approaches, allowing for the identification of key members of the epiphytic microbiota. Macroalgae host specific bacterial communities that largely differ from those occurring in the surrounding seawater (Weigel and Pfister 2019; Lu et al. 2023). Among bacteria associated with macroalgae, the bacterial phyla *Pseudomonadota* (mostly the classes *Alphaproteobacteria* and *Gammaproteobacteria*), *Bacteroidota*, *Verrucomicrobiota*, *Actinomycetota* and *Planctomycetota* are the most represented (Wahl et al. 2012; Hollants et al. 2013; Florez et al. 2017). At the genus level, the algal symbiont *Granulosicoccus* (*Gammaproteobacteria*) is commonly found as the most abundant taxon on diverse algal hosts (Singh and Reddy 2014), frequently reported to account for more than 25% of the whole community on kelp tissue (Brunet et al. 2021; Lemay et al. 2021; Ramírez‐Puebla et al. 2022; Weigel et al. 2022). It is now well established that bacterial community composition and density are shaped by biotic and abiotic factors and vary depending on biogeography, host tissue composition or seasonality (Lachnit et al. 2011; Egan et al. 2013; Lemay et al. 2018; Serebryakova et al. 2018; Weigel and Pfister 2019; Paix et al. 2021; Ramírez‐Puebla et al. 2022). Algae release a wide range of bioactive compounds with a broad spectrum of activities that participate in the control of bacterial colonisation (Saha and Fink 2022). It was reported that host taxonomy (Kuba et al. 2021; Chen et al. 2022), anatomy (Bengtsson et al. 2012; Weigel and Pfister 2019; Lemay et al. 2021), genetics (Wood et al. 2022), health (Fernandes et al. 2012; Marzinelli et al. 2015; Burgunter‐Delamare et al. 2023) or life cycle (Bengtsson et al. 2010; Glasl et al. 2021) influence its surface microbiota. Studies investigating seasonal variations in bacterial communities associated with healthy macroalgae have revealed dynamic patterns characterised by shifts in community composition and diversity (Lachnit et al. 2011; Burgunter‐Delamare et al. 2023). These shifts can often be linked to seasonal changes in environmental factors such as temperature, light or nutrient availability (Mancuso et al. 2016; Florez et al. 2019), which influence the growth and metabolic activity of both macroalgae and associated bacteria. Additionally, seasonal variations in macroalgal physiology, such as growth rates and reproductive cycles, can indirectly impact bacterial communities by altering the tissues and exudate composition (Pandey et al. 2022). Most of the works that characterised macroalgal‐associated microbiota mainly focused on a single algal species. Only a few recent studies compared algae from different phyla and of distinct chemical composition, revealing that algal phylum was a strong driver of epimicrobiota composition (Kuba et al. 2021) and identifying core taxa (Chen et al. 2022; Lu et al. 2023). Moreover, temporal dynamics of epiphytic bacterial communities depending on the algal host and type of tissue have been largely overlooked. In this study, the microbiota of three brown ( *Laminaria digitata* (Hudson) Lamouroux 1813, *Fucus serratus* Linnaeus 1753 and *Ascophyllum nodosum* (Linnaeus) Le Jolis 1863), one red ( *Palmaria palmata* (Linnaeus) Weber and Mohr 1805) and one green (*Ulva* sp. Linnaeus 1753) macroalgal species predominant in the English Channel were investigated throughout a year. This selection of species covers a large phylogenetic range and different intertidal zonation ( *A. nodosum* is found on the upper‐middle intertidal zone while *F. serratus* , *P. palmata* and *Ulva* sp. are in the lower intertidal zone and *L. digitata* in the subtidal zone (Evans 1957; Burel et al. 2022)). The sole use of metabarcoding amplicon sequencing has emerged as the preferred method to investigate host‐associated microbial communities. The resulting datasets are inherently compositional as they only allow analysis based on relative proportions of microbial taxa (Gloor et al. 2017). Therefore, an increase in abundance of one taxon can partly be caused by an equivalent decrease of another one, limiting the conclusions that can be drawn (Jian et al. 2020). Despite the statistical approaches applied to overcome misinterpretations of bacterial community structure (Gloor et al. 2017; Shelton et al. 2022), one of the most effective ways to accurately compare microbial communities across samples and to identify patterns that may be obscured when relying solely on relative abundance information, is to generate quantitative microbial abundance data by combining amplicon sequencing with the cell or gene copy density measurements. Several studies demonstrated an improved performance of quantitative approaches over compositional data (Jian et al. 2020; Lloréns‐Rico et al. 2021) and this has been recently implemented to examine human (Vandeputte et al. 2017; Barlow et al. 2020), freshwater (Props et al. 2017) or soil (Camacho‐Sanchez 2023) microbiomes. Here we coupled 16S rRNA gene amplicon sequencing with quantitative PCR to accurately estimate the abundance of the different taxa in macroalgal epiphytic communities. We aimed to (i) characterise the shared and specific patterns of epiphytic bacterial communities associated with co‐occurring macroalgal species and (ii) assess if seasonality equally impacts microbiomes from phylogenetically distant species.

## 2 Experimental Procedures

### 2.1 Sampling

The collection of epibiota from algal specimens was previously described in (Brunet et al. 2023). Briefly, the surface microbiota of intertidal algae was sampled monthly between February 2020 and January 2021 (no sampling in April and May 2020 due to the Covid‐19 pandemic). Microbiota were collected in triplicates using sterile flocked nylon swabs (Zymobiomics) on healthy specimens of the brown algae *L. digitata* (Ldig, 0.5–1 m long), *F. serratus* (Fser) and *A. nodosum* (Anod), the red alga *Palmaria palmata* (Ppal) and a green alga *Ulva* sp. (Ulva) at the Bloscon site (48°43′29.982″ N, 03°58′8.27″ W) in Roscoff (Brittany, France). The swabbed surface was standardised to 50 cm^2^. Based on previous evidence of strong tissue‐specific differences in the microbiota of *Laminariales* species (Ihua et al. 2020; Lemay et al. 2021; Burgunter‐Delamare et al. 2023), three different regions of *L. digitata* were sampled: the basal meristem (young tissue, hereafter LdigB), the medium frond (ca. 20 cm away from the meristem, hereafter LdigM) and the old frond (the blade tip, hereafter LdigO). Only two replicates were retrieved for LdigB in February and Ulva in March. Upon collection, swabs were immediately immersed in DNA/RNA Shield reagent (ZymoBiomics) on ice and stored at −20°C until DNA extraction. A total of 208 samples were collected (File S1), distributed between 70 different conditions (7 types of algal tissues at 10 different months).

### 2.2 Collection of Environmental Data

For collection of environmental data, we took advantage of a long‐term monitoring station located only 1.3 km from the algal sampling site (Service d'Observation en Milieu Littoral SOMLIT; http://www.somlit.fr; Estacade station). A set of 15 environmental parameters measured between February 2020 and January 2021 was used for correlation analyses. It includes surface seawater temperature (T, °C), salinity (S), dissolved oxygen (O, mL*L^−1^), pH, ammonium (NH_4_, μM), nitrate (NO_3_, μM), nitrite (NO_2_, μM), phosphate (PHO_4_, μM), silicate (SiOH_4_, μM), particulate organic carbon (COP, μg*L^−1^) and nitrogen (NOP, μg*L^−1^), suspended matter (MES, mg*L^−1^), ^15^Nitrogen (DN15, °/_°°_) and ^13^Carbon isotopes (DC13, °/_°°_) and Chlorophyll a (CHLA, μg*L^−1^). Data were downloaded from the SOMLIT database (Cocquempot et al. 2019) on April 7th, 2022 and are listed in File S1. Variables that were highly correlated to others were not used in further multivariate analyses (see below).

### 2.3 DNA Extraction and qPCR Assays

DNA extraction and qPCR assays were performed as in Brunet et al. (2023). Briefly, environmental DNA from swabs was extracted using the DNA/RNA Miniprep kit (ZymoBiomics) following the manufacturer's instructions and eluted in 50 μL RNase‐free water. DNA concentrations obtained from each sample with the QuantiFluor dsDNA System (Promega) kit are listed in File S1. All samples were normalised at 0.5 ng·μL^−1^ before qPCR and library preparation, except for three samples which had concentrations lower than 0.5 ng·μL^−1^ and were used without further dilutions. The number of total 16S rRNA gene copies was assessed using qPCR (primers targeting V6 region; 926F: 5′‐AAACTCAAAKGAATTGACGG‐3′/1062R: 5′‐CTCACRRCACGAGCTGAC‐3′ (De Bacchetti Gregoris et al. 2011)). The global predicted coverage of this primer pair is 94.1% of all bacterial sequences present in Silva SSU r138.1 (Silva Testprime with only one mismatch allowed, analysis performed in June 2024). Reactions (5 μL) were prepared using 1.5 μL of input DNA as matrix and 300 nM of each primer with the LightCycle 480 SYBR Green I Master kit (Roche), and analysed on a LightCycler 480 Instrument II (Roche). The total number of 16S rRNA gene copies per cm^2^ of algal surface (*N*) was calculated as follows:

$$N=\frac{n\times C\times V}{c\times v\times S}$$

where *n* is the number of 16S rRNA gene copies detected in the qPCR reaction, *C* is the original concentration of the DNA extract before normalisation (in ng·μL^−1^), *V* is the total volume of DNA extract (50 μL), *c* is the concentration of input DNA in the reaction (0.5 ng·μL^−1^ in most cases, see above), *v* is the volume of input DNA in the reaction (1.5 μL) and *S* is the total swabbed algal surface (50 cm^2^). qPCR results are listed in File S1, and MIQE information related to qPCR experiments is available in File S2.

### 2.4 Library Preparation, Sequencing and Sequence Processing

The V3–V4 region of the 16S rRNA gene was amplified using the primers S‐D‐Bact‐0341‐b‐S‐17 (5′CCTACGGGNGGCWGCAG 3′) and 799F_rc (5′CMGGGTATCTAATCCKGTT 3′), specifically designed to minimise plastid amplification in 16S rDNA metabarcoding analyses of alga‐associated bacterial communities (Thomas et al. 2020). Sequencing was conducted on a MiSeq paired‐end sequencing run (300 cycles × 2, Illumina, San Diego, CA, USA) as described previously (Thomas et al. 2020). 16S rRNA gene amplicon sequences are available at NCBI under BioProject PRJNA1135191. The R package *DADA2* (version 1.22.0, Callahan et al. 2016) was used to exclude primer sequences, filter and trim low‐quality sequences (truncLen = c(280, 210), trimLeft = c(10, 10), minLen = 150, maxN = 0, maxEE = c(2, 3), truncQ = 2), for denoising, paired‐read merging, inferring amplicon sequence variants (ASVs, i.e., unclustered exact nucleotide sequences of 16S rDNA reads (Glassman and Martiny 2018)), chimera removal and taxonomic assignment using the SILVA 138.1 reference database (Quast et al. 2013; Yilmaz et al. 2014). Default parameters were used unless otherwise specified. A total of 10,420 ASVs were retrieved (length 400–452 nucleotides).

### 2.5 Multivariate and Statistical Analyses

The R package *phyloseq* (version 1.38.0, McMurdie and Holmes 2013) was used for analysis using default parameters unless stated otherwise below. All sequences affiliated to the 16S rRNA gene from cyanobacteria (including chloroplasts), mitochondria and Eukaryota were removed (104 ASVs in total, see File S3 for details). Ten ASVs found both in negative controls (PCR molecular grade water) and in low frequency and abundance in algal samples (max. frequency 11 over 208 samples, max. relative abundance 1.5%) were considered as contaminants and removed from the dataset. Relative ASV abundance was multiplied by the total number of 16S rRNA gene copies quantified with qPCR for each sample, to obtain a table of abundance of each ASV that was used in all subsequent analyses. Differences in the coverage of qPCR vs. metabarcoding primer pairs and how this could affect the calculated results are discussed in File S3. To assess dissimilarities in community structure between algal tissues, the Bray–Curtis dissimilarity index (Bray et al. 1957) based on the ASVs absolute abundance table was calculated before non‐metric multidimensional scaling (NMDS). Permutational analysis of variance (PERMANOVA, 999 permutations) was calculated using the adonis function to discriminate groups of samples according to algal tissues or seasons (Anderson 2001), followed by multivariate pairwise comparisons using pairwise.perm.manova. Mean dispersions within groups were calculated using betadisper (999 permutations). To identify environmental parameters that may be associated with microbial community structure, we performed distance‐based redundancy analysis (dbRDA; Legendre and Anderson 1999) on the Bray–Curtis dissimilarity matrix with the dbrda function. Correlations between environmental parameters were tested using Pearson correlation tests (Figure S1). Variables highly correlated to at least one other parameter were removed prior to the analysis (pH, SiOH4, NOP, PO4, NO3, NO2, DC13). Only significant environmental factors were selected for dbRDA visualisation (ANOVA, *p* < 0.05). Months were grouped in 3 different seasons (Winter: Jan, Feb, Mar; Summer: Jun, Jul, Aug; Autumn: Sep, Oct, Nov, Dec) prior to PERMANOVA and betadisper analysis. Differential sequence abundance analysis between the 3 seasons (qPCR‐corrected counts from months of the same season were averaged) was performed at the ASV level using the GLM test with the ALDEx2 R package (Fernandes et al. 2014). To detect differential abundance, this test uses ratios among taxa, which are conserved regardless of whether data are relative or absolute.

Abundance of ASVs assigned to the same genus was pooled using the tax_glom function from the *phyloseq* R package. Upset plots were made using the R package ComplexUpset (version 1.3.3., Krassowski et al. 2020) at both the ASV and genus level. A standardised approach based on occupancy–redundancy distribution was applied to identify core genera on each algal tissue (Shade and Stopnisek 2019). Using a presence/absence matrix (1 for presence, 0 for absence), an index was calculated for each genus, as the sum of the occupancy term and the redundancy term for each month. The occupancy term is the replicate average of the presence/absence matrix. The redundancy term is equal to 1 if the genus is present in all three replicates and to 0 otherwise. The index was scaled to an upper limit of 1 and calculated separately for each algal tissue. Based on the distribution of index values (data not shown), we chose a lower limit of index = 0.65 for core genera. The number of ASVs shared between samples from the same brown algal species (“intra‐species”), between different brown algal species (“intra‐phylum”) and between brown algae and Ulva or Ppal (“inter‐phylum”) was compared using Kruskal–Wallis rank sum test followed by post hoc pairwise Wilcoxon test with Benjamini‐Hochberg corrections of *p* values.

### 2.6 Phylogenetic Analysis

Phylogenetic analysis of ASVs affiliated to the genus *Granulosicoccus* was conducted using phylogeny.fr (Dereeper et al. 2008). Since the dataset comprised a total of 464 *Granulosicoccus* ASVs, we focused our analysis on the most abundant and persistent ASVs (representing at least 5% of the *Granulosicoccus* community in at least 5 samples; 28 ASVs). For references, we included 16S rRNA gene sequences from 14 cultured *Granulosicoccus* strains and seven uncultured clones retrieved from Genbank (see Figure 4 for accession numbers). The 16S rRNA gene from *Sulfuriflexus mobilis* aks1^T^, another member of the *Granulosicoccaceae* family, was used as an outgroup (Kojima and Fukui 2016). A total of 50 sequences were aligned using Muscle (full mode). The resulting alignment (1607 positions) was curated using Gblocks to keep the V3–V4 region of the 16S rRNA gene amplified with primers S‐D‐Bact‐0341‐b‐S‐17/799F_rc and eliminate poorly aligned positions. The curated alignment (434 positions) was used for neighbour‐joining tree reconstruction with the K2P substitution model and 10,000 bootstraps. The tree was visualised using iTOL (Letunic and Bork 2024). The sequence of the outgroup *S. mobilis* aks1^T^ was used to root the tree.

## 3 Results

The structure and dynamics of the bacterial community associated with five phylogenetically distant macroalgae from a temperate rocky shore have been investigated over 1 year. The ASV relative abundance was multiplied by the total number of 16S rRNA gene copies to consider absolute taxa count and obtain a more precise understanding of the algal epibacteriome structure.

### 3.1 Algal Host as a Major Driver of Epiphytic Bacterial Community Composition

**FIGURE 1:**
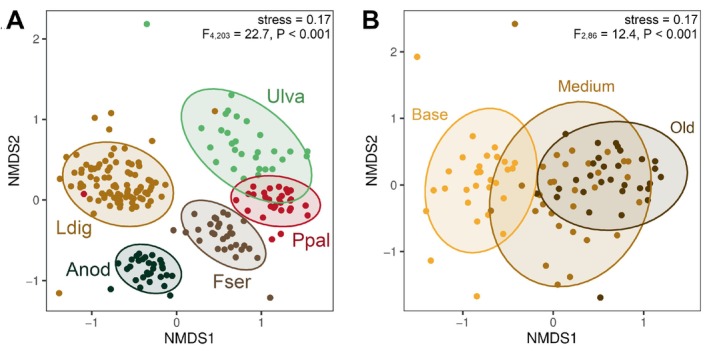
NMDS ordination plots of abundance data of all samples (A) or *L. digitata* samples (B), based on Bray–Curtis dissimilarity. 95% confidence ellipses for a multivariate *t*‐distribution are depicted for the different type of algal tissues (either algal species or *L. digitata* blade part). PERMANOVA *F*‐statistic and *p* value are displayed.

The quantified number of bacterial 16S rRNA gene copies (File S1) spanned more than two orders of magnitude, from a minimum of 1.04 × 10^5^ to a maximum of 5.02 × 10^7^ 16S rRNA gene copies cm^−2^. This proxy for total bacterial abundance was relatively homogeneous for all algal species, with year‐round averages ranging between 7 × 10^6^ and 1 × 10^7^ 16S rRNA gene copies cm^−2^. Bacterial abundance varied significantly along the year (2‐way ANOVA, ‘sampling month’ effect *F*_9,158_ = 5.0, *p* < 0.001) but not across macroalgal species (*F*_4,158_ = 0.8, *p* = 0.531) and with no interactions between the two factors (*F*_36,158_ = 1.1, *p* = 0.3). The structure of the epiphytic bacterial communities was strongly impacted by the algal host (Figure 1A; PERMANOVA, *F*_4,203_ = 22.7, *p* < 0.001), showing that communities from different algal species sharing the same habitat are distinct from one another. Only 1% of the total number of ASVs (103 out of 10,243) were detected at least once on each of the 5 sampled algal species (Figure S2B). Sixty‐six percent (6801 ASVs) were only found in one sample, suggesting a high variation of the microbiota at the sample level. Despite their closer phylogenetic distance, brown algae (Ldig, Anod and Fser, all belonging to the class Phaeophyceae) did not share more ASVs between them than with green (Ulva) and red (Ppal) algae (Figure S3). This was not due to high sample‐to‐sample variation, since samples within the same algal species shared significantly more ASVs than samples from different algal species (Figure S3, Kruskal–Wallis test, *p* = 0.023). Besides host taxonomy, the age of algal tissue also influenced the surface density and composition of the microbiota. Indeed, the younger meristematic tissues of *L. digitata* (Ldig‐base) consistently harboured fewer 16S rRNA gene copies than the medium frond (Ldig‐medium, paired *t*‐test, *p* = 0.002) and the older apical tissues (Ldig‐old, paired *t*‐test, *p* < 0.001). Multivariate analysis also separated Ldig‐base epibacterial communities from Ldig‐medium and Ldig‐old (Figure 1B; PERMANOVA, *F*_2,86_ = 12.4, *p* < 0.001).

**FIGURE 2:**
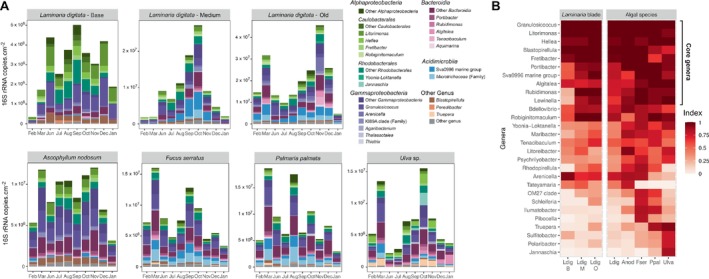
(A) Estimated abundance of the main bacterial genera. Only genera representing more than 5% (triplicate average) of the communities for at least one condition (i.e., at one time point for one algae) are shown. (B) Approach to identify core macroalgal genera. Indices are calculated based on abundance‐occupancy distributions (see Methods for index calculation). Only genera that have an index > 0.5 on at least one type of tissue are represented. For the Ldig column, the index is an average of the indices calculated on the base (B), medium (M) and old (O) Ldig tissues.

*Gammaproteobacteria*, *Alphaproteobacteria*, *Bacteroidia, Acidimicrobiia and Planctomycetes* were the most abundant bacterial classes on all algae, representing on average 93%–98% of the communities, depending on the algal host (Figure S4). After pooling the ASVs by genus, we observed that 28% of all genera (93 out of 331) were detected at least once on each algal species, suggesting they might be ubiquitous algal epibionts shared between all algae (Figure S2A and File S4). These generalists were mainly *Bacteroidia* (including *Flavobacteriales*), *Gammaproteobacteria* and *Alphaproteobacteria* and represented on average 50%–75% of all sequences depending on algae. An occupancy‐redundancy index was calculated following Shade and Stopnisek (2019) method to identify core genera that were consistently found associated with the five studied algal species all year round (Figure 2B). Genera that had an index > 0.65 on all algae were defined as core genera: *Granulosicoccus* (*Gammaproteobacteria*), *Litorimonas*, *Hellea*, *Fretibacter (Alphaproteobacteria)*, *Portibacter*, *Algitalea*, *Rubidimonas*, *Lewinella (Bacteroidota)*, *Blastopirellula* (*Planctomycetota*) and Sva0996 marine group (*Actinomycetota*). These represented only 3% of all genera, yet accounted for 46% of the bacterial abundance on average. The genus *Granulosicoccus* was the only one to have an index equal to 1 on all algal tissues, meaning it was present in all 208 samples (see below). On the contrary, some genera were specifically associated with particular algae. Especially, the genus *Arenicella* (*Gammaproteobacteria*) was strongly associated with brown algae all year round (index > 0.78, 2.8 × 10^5^ copies cm^−2^ on average) while scarce on Ppal (index = 0.27, 3.4 × 10^4^ copies cm^−2^) and Ulva (index = 0.2, 4.1 × 10^3^ copies cm^−2^). The opposite was observed for *Truepera* (*Deinococcota*; index < 0.3 on brown algae, > 0.9 on the red and green algae). Some genera were only found strongly associated (index > 0.8) with one algal species, namely *Tateyamaria* (*Alphaproteobacteria*) on Anod, *Pibocella* (*Bacteroidota*) on Fser, *Sulfitobacter (Alphaproteobacteria)*, *Polaribacter (Bacteroidota)* and *Jannaschia* (*Alphaproteobacteria*) on Ulva. Moreover, important differences were observed between the different parts of the Ldig frond (Figure 2). In particular, members of the Sva0996 marine group and of the family *Microtrichaceae* (*Actinomycetota*) accounted on average for only 3 × 10^4^ 16S rRNA gene copies cm^−2^ (1%) on the meristem compared to 2 × 10^6^ (15%) and 4 × 10^6^ (21%) on the medium and old frond respectively. These taxa were also overrepresented on Fser (2 × 10^6^, 21%) and Ppal (2 × 10^6^, 20%).

### 3.2 Seasonal Succession of Epiphytic Bacterial Communities

The number of 16S rRNA gene copies cm^−2^ varied differently through seasons depending on the algal host, and no common pattern to all algae could be highlighted (Figure 2). The number of total bacteria tended to reach a peak in late summer/autumn on Ldig, Fser, Ppal and Ulva. Ldig‐old, Anod, Fser, Ppal and Ulva samples from March 2020 showed higher bacterial abundances than neighbouring months (February and June). The absence of data in April and May (COVID lockdown) prevents us from assessing if this abundance peak was an isolated event caused by specific environmental conditions in March or if it was characteristic of the entire spring season.

Globally, the number of observed ASVs on each algal tissue did not significantly differ through the year (Figure S5), except for individual comparisons June/October for Ldig‐medium, June/December for Ldig‐old and June/September for Ulva.

**FIGURE 3:**
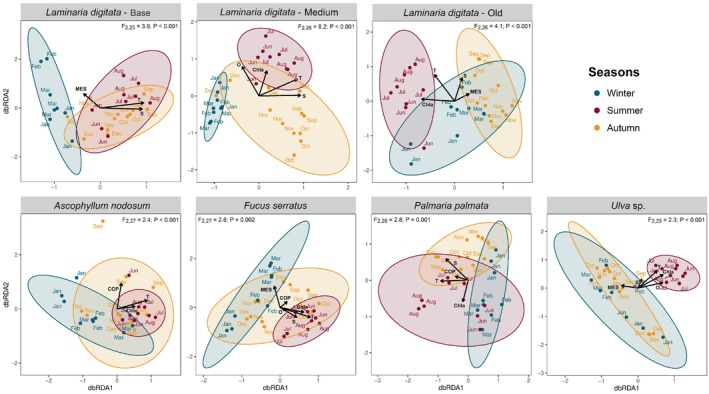
The db‐RDA plots with vectors representing significant contextual predictors. 95% confidence ellipses for a multivariate *t*‐distribution are depicted for the different seasons. PERMANOVA *F*‐statistic and *p* value are displayed. ChlA: chlorophyll A; COP: particulate organic carbon; MES: suspended matter; O: dissolved oxygen; S: salinity; T: temperature.

dbRDA and PERMANOVA (Figure 3) showed that all algal tissues displayed clear dissimilarities between seasons. For all algae, the three identified seasons (winter, summer and autumn) were all distinct from one another (*p* < 0.05; except between winter and autumn with Anod and Ulva). The analysis suggested that these seasonal variations might be partly explained by some environmental parameters, especially seawater temperature, salinity and chlorophyll A concentration, which were identified as significant contextual predictors.

Differential abundance analysis at the ASV level showed that no single ASV was similarly impacted by seasons across all algae (File S5). This might be partly explained by the low proportion of ASVs present on all algae, as mentioned above. We therefore focused on variations found for each algal species separately. Anod‐associated communities were the least impacted by seasons with only 9 ASVs differentially abundant (File S5). For Ldig, bacterial communities' seasonal dynamics varied depending on the blade part. The basal part exhibited fewer variations than the older ones (15 differentially abundant ASVs compared to 55 and 92 on medium and old fronds, respectively). On the basal tissues, 2 ASVs from the genus *Peredibacter* (*Bdellovibrionota*) were more abundant during summer months (3 × 10^4^ 16S rRNA gene copies cm^−2^, against 2 × 10^2^ in winter and 6 × 10^3^ in autumn, respectively) and 3 ASVs from the family *Rhodobacteraceae* (*Alphaproteobacteria*) less abundant in winter. The most outstanding abundance variations on the older tissues were observed for ASVs from the classes *Acidimicrobiia* (Sva0996 marine group and the family *Microtrichaceae*) and *Bacteroidia* (orders *Chitinophagales* and *Flavobacteriales*), which peaked in autumn on the medium frond and in autumn and winter at the tip part. For Fser, the community succession was characterised by a significantly higher abundance of *Micavibrionales* (*Alphaproteobacteria*) in summer (2 × 10^4^ 16S rRNA gene copies cm^−2^, against 0 in winter and 2 × 10^3^ in autumn). Also, the abundance of 28 *Chitinophagales* (*Bacteroidota*) ASVs significantly varied between seasons but exhibited distinct abundance patterns. For Ppal, only a few variations were observed (24 differentially abundant ASVs). The most notable succession was a peak of the *Aquimarina* (*Flavobacteriales*) population in winter (1 × 10^5^ 16S rRNA gene copies cm^−2^ compared to none in summer and 4 × 10^3^ autumn). For Ulva, summer communities displayed high specificity. Thirty‐one ASVs were differentially abundant; in particular, members of the genus *Truepera* (*Deinococcales*), of the families *Micotrichaceae* (*Actinomycetota*) and *Saprospiraceae* (*Bacteroidota*) exhibited a sharp decrease in summer while alphaproteobacteria from orders *Rhodobacterales* and *Caulobacterales* (*Litorimonas* and *Fretibacter*) observed an opposite trend.

### 3.3 *Granulosicoccus* Are Ubiquitous Members of Macroalgal Epibiota

**FIGURE 4:**
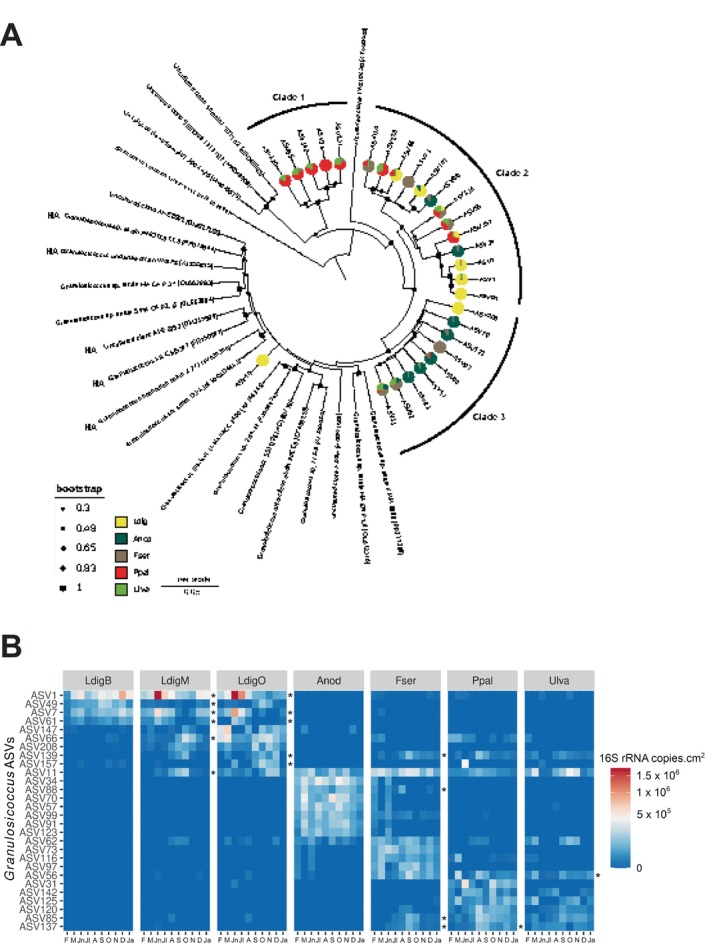
(A) Phylogenetic tree of the main 16S rRNA ASVs affiliated to *Granulosicoccus* and reference sequences (Genbank accession in brackets). Pie charts represent year‐round average abundance of each ASV on different algal species. HA: host‐associated. (B) Heatmap of the abundance of the main ASVs assigned to the genus *Granulosicoccus*. Only *Granulosicoccus* ASVs representing at least 5% of the *Granulosicoccus* community in at least 5 conditions (out of 70) are displayed. Abundances of triplicates were averaged and square root transformation was applied. Asterisks on the right panel of each alga denote differentially abundant ASVs through seasons. A: August; D: December; F: February; Ja: January; Jl: July; Jn: June; M: March; N: November; O: October; S: September.

The genus *Granulosicoccus* (*Gammaproteobacteria*) was the only one present in all 208 samples, covering the 5 different algal species and the 10 sampling months. It was also the most abundant genus, representing 15.4% of the communities on average (up to 42% on Ldig‐base) and accounting for 1.2 × 10^6^ copies cm^−2^ on average (up to 7.7 × 10^6^ copies cm^−2^ on Ppal). It comprised 464 different ASVs, the highest number of ASVs for all genera in our dataset, indicating a large micro‐diversity. Half of these ASVs (236) were only found in one condition (i.e., on one type of tissue at one specific time point). Phylogenetic analysis of the most abundant and persistent *Granulosicoccus* ASVs (representing at least 5% of the *Granulosicoccus* community in at least 5 conditions) separated them into three main clades and indicated that they cluster separately from the 4 described species (Figure 4). Clades 2 and 3 clustered with reference sequences from cultured *Granulosicoccus* strains and uncultured clones. This notably included other marine host‐associated strains, retrieved from the brown alga *Undaria*, the red alga *Gracilaria*, the seagrass *Zostera marina* , sponge and plankton (Kurilenko et al. 2010; Park et al. 2014; Heins and Harder 2023). Clade 1 comprised 5 ASVs that clustered with 3 uncultured clones from microbial mats of the Southern Mariana Trough. In addition, all ASVs were not equally distributed across the different algal species. Only two closely related *Granulosicoccus* ASVs were consistently detected on all algal species (ASV11 and ASV62). Most of the ASVs were specific or strongly linked to a unique algal species, for example, ASV1, ASV7, ASV61, ASV208 and ASV49 to Ldig, ASV34, ASV70, ASV123, ASV91 and ASV57 to Anod, ASV73 to Fser and ASV31 to Ppal (Figure 4A). While clade 2 encompassed ASVs detected on multiple algae, clade 1 and clade 3 comprised ASVs more abundant on red and brown algae, respectively. *Granulosicoccus* ASVs associated with medium and old Ldig tissues exhibited stronger significant seasonal variations than *Granulosicoccus* ASVs associated with the other algal species (Figure 4B). Moreover, even ASVs found on the same tissue displayed distinct variation patterns, for example, for Ldig‐medium where ASV1, 7 and 61 peaked in summer and ASV66, 208 and 139 in autumn.

## 4 Discussion

Next‐generation sequencing methods have become indispensable tools in microbial ecology, opening new avenues for studying microbial communities. However, these approaches are limited, as they yield relative abundance profiles of microbial taxa, which may not accurately reflect true variations in the actual taxon abundances in the environment (Gloor et al. 2017; Lloréns‐Rico et al. 2021). In this study, we estimated actual ASV abundance by multiplying the ASV relative abundance matrix by the corresponding number of 16S rRNA gene copies estimated through qPCR (Barlow et al. 2020; Jian et al. 2020). This approach has been previously applied to soil microbiota (Lou et al. 2018; Azarbad et al. 2022), revealing that quantification provides a more comprehensive understanding of bacterial community dynamics than compositional data, which are more prone to false‐positive changes. Absolute abundance of bacterial taxa associated with macroalgae has been previously characterised, but focusing only on a few targeted taxa using specific qPCR primers or FISH probes (Ramírez‐Puebla et al. 2022; Brunet et al. 2023). Here we implemented this method to quantitatively characterise the whole macroalgal epimicrobiota composition for the first time, aiming to assess true taxa variations across different algal hosts and seasons. Although providing valuable improvements over sole amplicon sequencing to estimate absolute abundance of different taxa, coupling with qPCR still has some limitations. At least three limitations are common with the metabarcoding‐only approach: (1) potential saturation of the swabs during sampling that could lead to underestimate bacterial abundance on densely colonised algal surfaces; (2) variation in extraction efficiencies depending on algal tissue composition (e.g., polysaccharide content); (3) unequal lysis of different bacterial taxa. Here, we used flocked nylon swabs, previously shown to outperform other swab types in terms of collection efficiency and DNA recovery from microbiomes (Bruijns et al. 2018; Wise et al. 2021). The large variation in DNA concentrations retrieved from swabs (minimum 0.11 ng·μL^−1^, median 7.57 ng·μL^−1^, maximum 41.46 ng·μL^−1^, 92% of samples below 24 ng·μL^−1^, File S1) further suggests that if saturation occurred, it only affected a limited number of samples. Moreover, DNA recovery from swabs did not differ depending on algal species (on average Anod: 9.3 ng·μL^−1^; Fser 8.9 ng·μL^−1^; Ldig 10.3 ng·μL^−1^; Ppal 10.6 ng·μL^−1^; Ulva 6.9 ng·μL^−1^, File S1), suggesting tissue composition did not largely affect extraction efficiency. An additional bias lies in the use of different sets of primers for qPCR and amplicon sequencing (see File S3). Indeed, qPCR requires short amplicon size for optimal efficiency and specificity (< 200 bp, Debode et al. 2017), whereas optimal primers for 16S metabarcoding cover a region of at least approximately 450 bp after merging, to encompass both conserved and hypervariable regions.

### 4.1 Common Core of the Epiphytic Microbiome

The large number of samples collected in this study, encompassing 7 types of algal tissues among 5 algal species at 10 sampling months, allows for the identification of a core epiphytic microbiome that would be consistently found on algae all year round. The identification of these core microbes is crucial as they likely have a more substantial impact on the host's biology than other members of the microbiome (Neu et al. 2021). Here, the core microbiota was identified based on occupancy and redundancy criteria as recently used in soil microbial ecology studies (e.g., Hodgson et al. 2024). The identified core genera, especially *Granulosicoccus*, *Litorimonas* and *Hellea*, concur with previous works that revealed the preponderance of these taxa at the surface of macroalgae (Weigel and Pfister 2019; Lemay et al. 2021; Wood et al. 2022; Burgunter‐Delamare et al. 2023; Lu et al. 2023). *Granulosicoccus* was the dominant genus on all algae, both in terms of presence/absence and abundance. The predominance of *Granulosicoccus* on the surface of other brown algae was previously suggested using relative compositional data (e.g., Lemay et al. 2021; Weigel et al. 2022; Burgunter‐Delamare et al. 2023) and quantitative cell counts using FISH probes (Ramírez‐Puebla et al. 2022). Here, we quantify this ubiquity on a larger temporal scale and on other algal species, extending this observation to red and green algae. Using an estimate of 3 rRNA operons per cell (as seen in the complete reference genome of *Granulosicoccus antarcticus* (Kang et al. 2018)), the maximum observed density of 7.7 × 10^6^ 16S rRNA gene copies cm^−2^ on the red algae *P. palmata* would correspond to ca. 2 million Granulosicoccus cells cm^−2^. Using FISH counts on the kelp *Nereocystis luetkeana* in summer, Ramírez‐Puebla et al. (2022) reported 5.5 × 10^4^ and 6.5 × 10^6^ *Granulosicoccus* cells cm^−2^ on basal and old tissues, respectively. Comparable values can be inferred from our dataset on the basal and old tissues of the kelp *L. digitata* in summer (5 × 10^5^ and 2.3 × 10^6^ *Granulosicoccus* cells cm^−2^, respectively), confirming qPCR correction of metabarcoding compositional data is a useful approach to estimate absolute abundances of specific taxa. *Granulosicoccus* sequences were scattered into multiple ASVs that fall within distinct clades whose abundance patterns across algal hosts and seasons differ. These clades exhibit distinct tissue specificity and could be characterised either as generalist (clade 2) for containing taxa colonising all types of tissue or specialist for clades 1 and 3 that encompass taxa that are more specific to particular hosts. These data indicate that ASVs from the same genus colonise distinct ecological niches and underscore important genetic and functional diversity within core bacterial genera associated with macroalgae. Similar observations were made in recent studies where different genomes of core epiphytic genera, especially *Granulosicoccus*, were differentially abundant at the surface of the bull kelp *N. luetkeana* depending on its location (Weigel et al. 2022) or on *Fucus* sp. depending on season and geographical location (Park et al. 2022). The analysis of *Granulosicoccus* genomes revealed strong abilities for an epiphytic lifestyle that would allow fast colonisation and stable associations with the host. In particular, genes involved in motility and chemotaxis, B12 vitamin biosynthesis, DMSP metabolism, transport and utilisation of sugars were found abundant in *Granulosicoccus* genomes (Kang et al. 2018; Weigel et al. 2022).

### 4.2 Host Algal Tissue Is a Major Driver of Epiphytic Community Composition

Our study revealed that the type of algal tissue, whether it is different algal species or different blade parts, was a stronger driver of epiphytic community composition than seasonality. This observation is consistent with previous reports stating that host characteristics may have a greater impact on shaping the structure of macroalgae‐associated microbial communities than environmental variables (Marzinelli et al. 2015; Burgunter‐Delamare et al. 2023). The chemical composition of algal tissues, including concentrations of proteins, minerals, lipids or sugars, varies depending on algal taxonomy (Mišurcová 2011) or tissue age (Küpper et al. 1998). In particular, both iodine and polyphenols are known to be important algal defence compounds involved in the regulation of microbial colonisation (e.g., Cosse et al. 2009; Besednova et al. 2020). Therefore, one explanation for the inter‐host microbiome dissimilarities observed in this study could be specific variations in the content of these compounds within or at the surface of algal tissues, although we did not measure them directly in this study. Indeed, in previous publications, polyphenol content was reported to be 10–20 times higher in *A. nodosum* and *F. serratus* compared to the other algae collected in the same study (Pandey et al. 2022). Species‐specific differences were also reported for iodine, as its concentration was shown to be approximately 5 and 20 times higher in *L. digitata* compared to *A. nodosum* or *F. serratus* and *P. palmata* or *Ulva* sp., respectively (Nitschke et al. 2018). Such chemical variations were also observed across seasons but were much less pronounced than between algal species (Nitschke et al. 2018; Pandey et al. 2022). Therefore, if these compounds are drivers of community composition, their limited annual variations within the tissues could partly explain why seasons had a smaller impact on community composition than algal hosts. Interestingly, microbiota composition similarities were not higher between brown algae ( *L. digitata* , *A. nodosum* and *F. serratus* ) than with the red or green algae. Similarly, no common pattern was found among species inhabiting the same intertidal zone. Then, despite the important discrepancies observed in the microbiota of distinct algal species, neither higher taxonomic level (algal phylum) nor algae location on the foreshore appeared to be strong community drivers, corroborating observations made by Chen et al. 2022 and Selvarajan et al. 2019. Nevertheless, the abundance of certain taxa displayed important differences between algal phyla; *Arenicella* was consistently associated only with brown algae, whereas *Truepera* was consistently found only with red and green algae. *Truepera* phylum specificity was also reported in (Lu et al. 2023) with different algal species.

This work shows that different parts of the *L. digitata* blade support distinct bacterial communities and that total bacteria abundance and diversity are higher on older tissues, in accordance with studies carried out on the same species (Ihua et al. 2020) and on other kelp species (Bengtsson et al. 2012; Weigel and Pfister 2019; Lemay et al. 2021; Ramírez‐Puebla et al. 2022; Burgunter‐Delamare et al. 2023). Moreover, the specific colonisation of older *L. digitata* tissues by members of the Sva0996 marine group, in comparison to the meristem, concurs with the one observed in (Brunet et al. 2021) where the abundance of this taxon was linked with tissue decomposition and degradation. Then, the presence of such taxa on old damaged tissues might be related to their capacity to use complex algal‐derived organic matter such as polysaccharides (Brunet et al. 2022).

Seasonal variations of the surface microbiota have received unequal attention depending on the algal host. To our knowledge, to date, there is no report of these fluctuations on *Ascophyllum* sp. or *Palmaria* sp., while a few studies were conducted on *Fucus* sp. (Lachnit et al. 2011; Park et al. 2022), *Ulva* sp. (Tujula et al. 2010; Lachnit et al. 2011) and *Laminaria* sp. (Corre and Prieur 1990; Bengtsson et al. 2010; Brunet et al. 2021). Seasonal microbial variations are known to be influenced by a combination of biotic and abiotic factors. Here we show that seasons affect epiphytic community composition for all algae but in different ways. Community richness decreased in summer for *L. digitata* and *Ulva* sp. Temperature and salinity were environmental parameters that could explain the observed seasonal discrepancies. Seawater temperature shapes microbial community variations; in particular, it was already shown that increasing temperatures lead to a decrease in community richness on kelp (Paix et al. 2021) and in seawater (Sunagawa et al. 2015). Salinity was also reported as an important structuring variable of *Ulva*‐associated bacterial communities (Van der Loos et al. 2023). Anod‐associated microbiota was the most stable across seasons among all five algae. *A. nodosum* is known to observe monthly epidermal shedding, a way for longer‐lived marine macroalgae to rid themselves of epibionts via the removal of the outer cell layer of the thallus (Halat et al. 2015; Garbary et al. 2017). Therefore, it is likely that the newly exposed thallus, free of epibionts, does not present any alterations or damages and is monthly colonised by the same early bacterial epibionts.

This study, through the extensive number of collected samples and the analysis of absolute taxa abundance, revealed true microbiota variations based on hosts and seasons, providing novel insights into the structure and fluctuations of bacterial communities associated with macroalgae. Future studies should focus on deciphering the ecological and metabolic functions of the identified core epiphytic taxa to gain further insights into epiphyte–host interactions.

## Author Contributions

**Maéva Brunet:** investigation, writing – original draft, visualization, writing – review and editing, formal analysis. **Nolwen Le Duff:** investigation, writing – review and editing. **Tristan Barbeyron:** supervision, writing – review and editing. **François Thomas:** conceptualization, investigation, funding acquisition, writing – original draft, visualization, writing – review and editing, formal analysis, project administration, supervision.

## Conflicts of Interest

The authors declare no conflicts of interest.

## Supporting information


**Figure S1.** Correlation matrix (Pearson coefficients) between environmental parameters. Pearson coefficients are indicated in black. Only significant correlations are shown (Benjamini–Hochberg adjusted *p* values < 0.05).

**Figure S2.** Upset plots made at the genera (A) and ASVs (B) level. The intersection size represents the number of genera/ASVs common to algae marked with a full black circle. Set size represents the total amount of genera/ASVs present on each alga. (A) Upset plot of the 331 bacterial genera found in the algal microbiota. Taxonomy is displayed at the class level for the genera shared by all algae (left bar) and the ones specific to one algal species (five last bars on the right). (B) Upset plot of the 10,243 ASVs found in the algal microbiota.

**Figure S3.** Number of ASVs shared between samples from the same brown algal species (intra‐species), between different brown algal species (intra‐phylum) and between brown algae and Ulva or Ppal (inter‐phylum). A Kruskal–Wallis rank sum test was performed to compare the number of shared ASVs in each three groups (*p* = 0.023), followed by post hoc pairwise Wilcoxon test with Benjamini‐Hochberg correction for which *p* values are shown on the graph.

**Figure S4.** Estimated abundance of the main bacterial classes. Only classes representing more than 3% (triplicate average) of the communities for at least one condition (i.e., at one time point for one algae) are shown.

**Figure S5.** Fluctuation of the number of observed ASVs. When significant ANOVA results were found (*p* < 0.05), a post hoc Tukey HSD test was calculated. Accordingly, different letters indicate significant differences between sampling months.

**File S1.** Characteristic of the 208 samples of macroalgal epibiota collected during this study.

**File S2.** Details of the qPCR assay on the environmental samples, following the Minimum Information for publication of Quantitative real‐time PCR Experiments (MIQE) guidelines.

**File S3.** Differences in primer coverage.

**File S4.** List of the 331 assigned taxonomic genera. The number of samples in which they were found is indicated for each alga.

**File S5.** List of differentially abundant ASVs through seasons. A red excel cell indicates the abundance of the ASV varies significantly through seasons on the associated algal tissue. The different sheets named after each algal tissue display the season average abundance of the differentially abundant ASVs. The genus (or the deepest known taxonomic level) assigned to each ASV is mentioned.

## Data Availability

The data that support the findings of this study are openly available in NCBI Short Read Archive under BioProject ID PRJNA1135191, and in article Supporting Information.
