# Supplementary figures and images for: Year‐Round Quantification, Structure and Dynamics of Epibacterial Communities From Diverse Macroalgae Reveal a Persistent Core Microbiota and Strong Host Specificities
Source: Environ Microbiol Rep. 2025 Mar 12;17(2):e70077. doi: 10.1111/1758-2229.70077 (PMC11903338; doi:10.1111/1758-2229.70077)

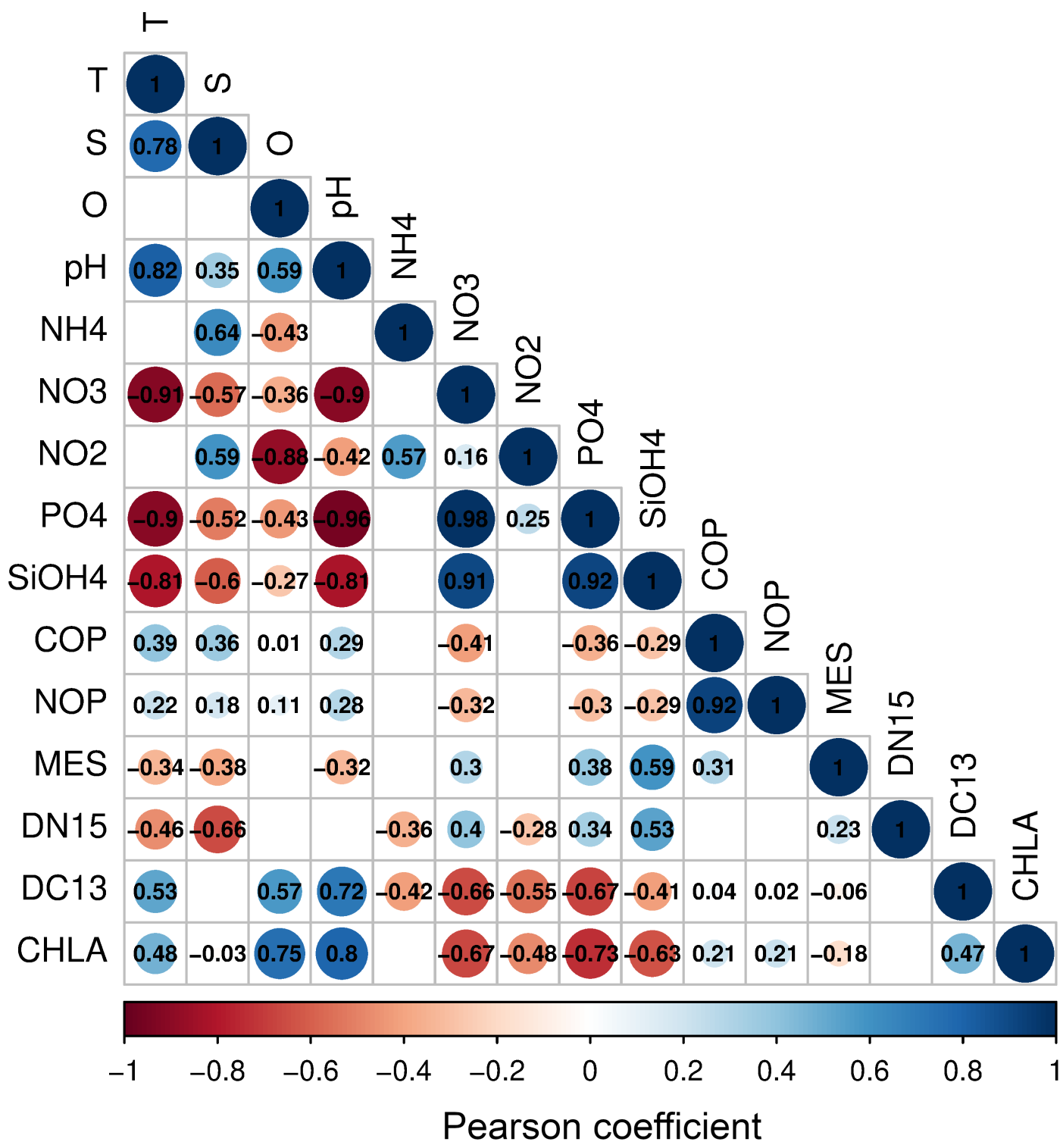

Supplement: Supplementary file 1 — Figure S1. Correlation matrix (Pearson coefficients) between environmental parameters. Pearson coefficients are indicated in black. Only significant correlations are shown (Benjamini–Hochberg adjusted p values < 0.05). [file EMI4-17-e70077-s009.pdf]

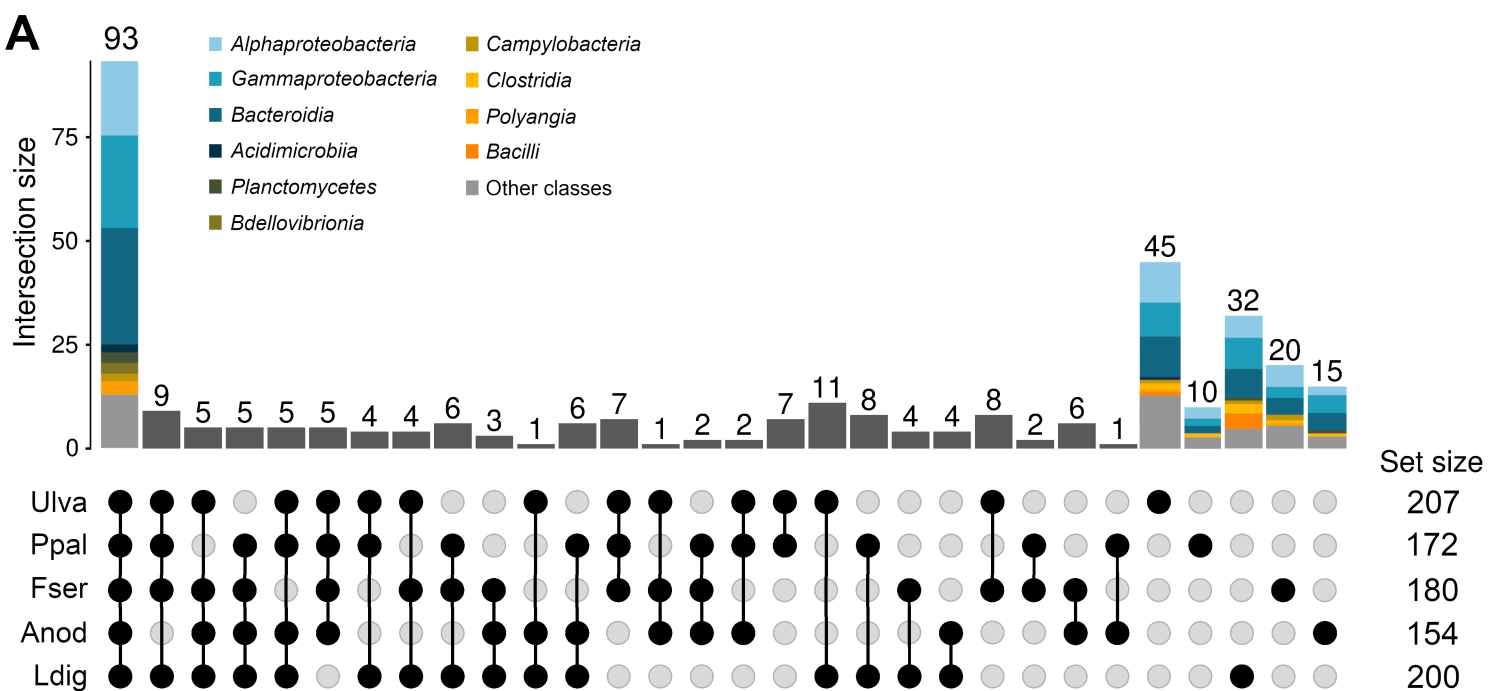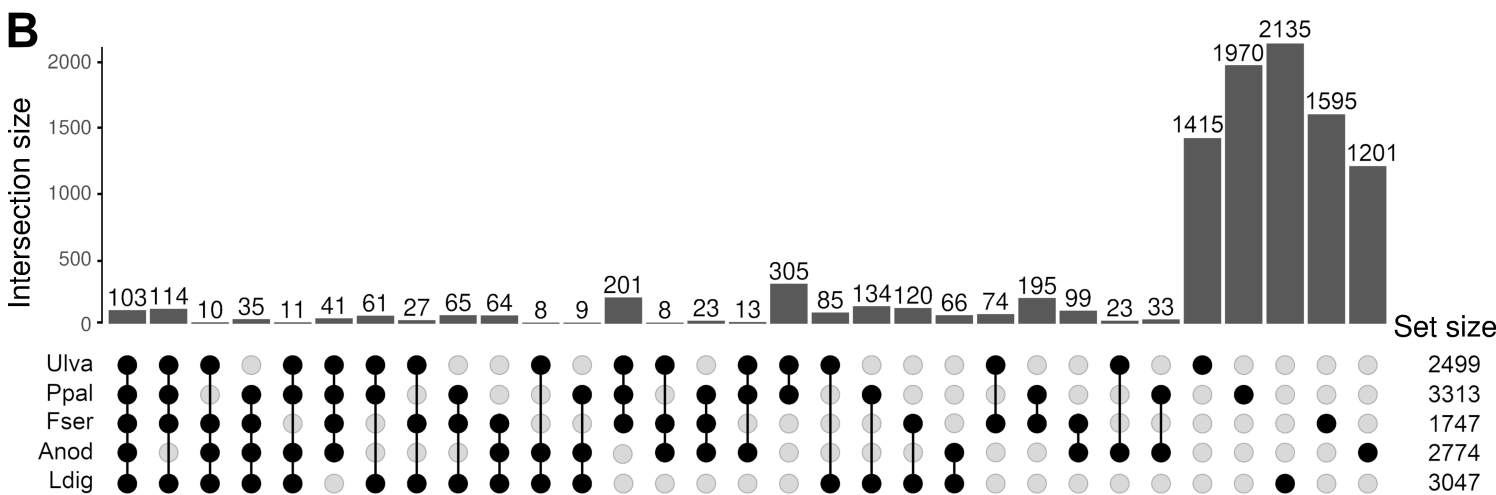

Supplement: Supplementary file 2 — Figure S2. Upset plots made at the genera (A) and ASVs (B) level. The intersection size represents the number of genera/ASVs common to algae marked with a full black circle. Set size represents the total amount of genera/ASVs present on each alga. (A) Upset plot of the 331 bacterial genera found in the algal microbiota. Taxonomy is displayed at the class level for the genera shared by all algae (left bar) and the ones specific to one algal species (five last bars on the right). (B) Upset plot of the 10,243 ASVs found in the algal microbiota. [file EMI4-17-e70077-s001.pdf]

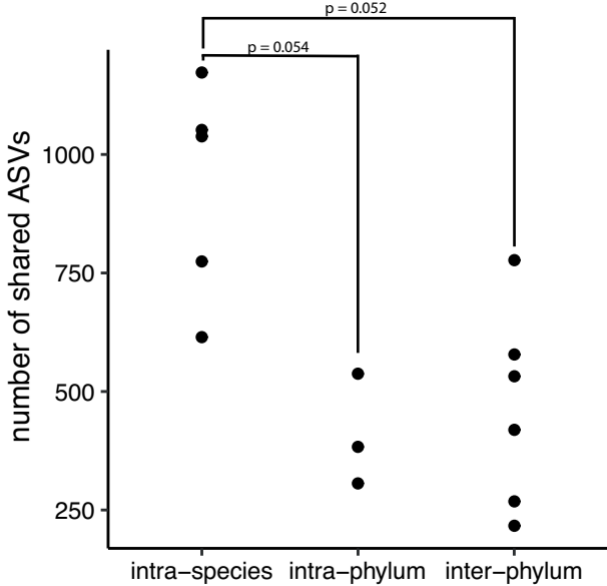

Supplement: Supplementary file 3 — Figure S3. Number of ASVs shared between samples from the same brown algal species (intra‐species), between different brown algal species (intra‐phylum) and between brown algae and Ulva or Ppal (inter‐phylum). A Kruskal–Wallis rank sum test was performed to compare the number of shared ASVs in each three groups (p = 0.023), followed by post hoc pairwise Wilcoxon test with Benjamini‐Hochberg correction for which p values are shown on the graph. [file EMI4-17-e70077-s005.pdf]

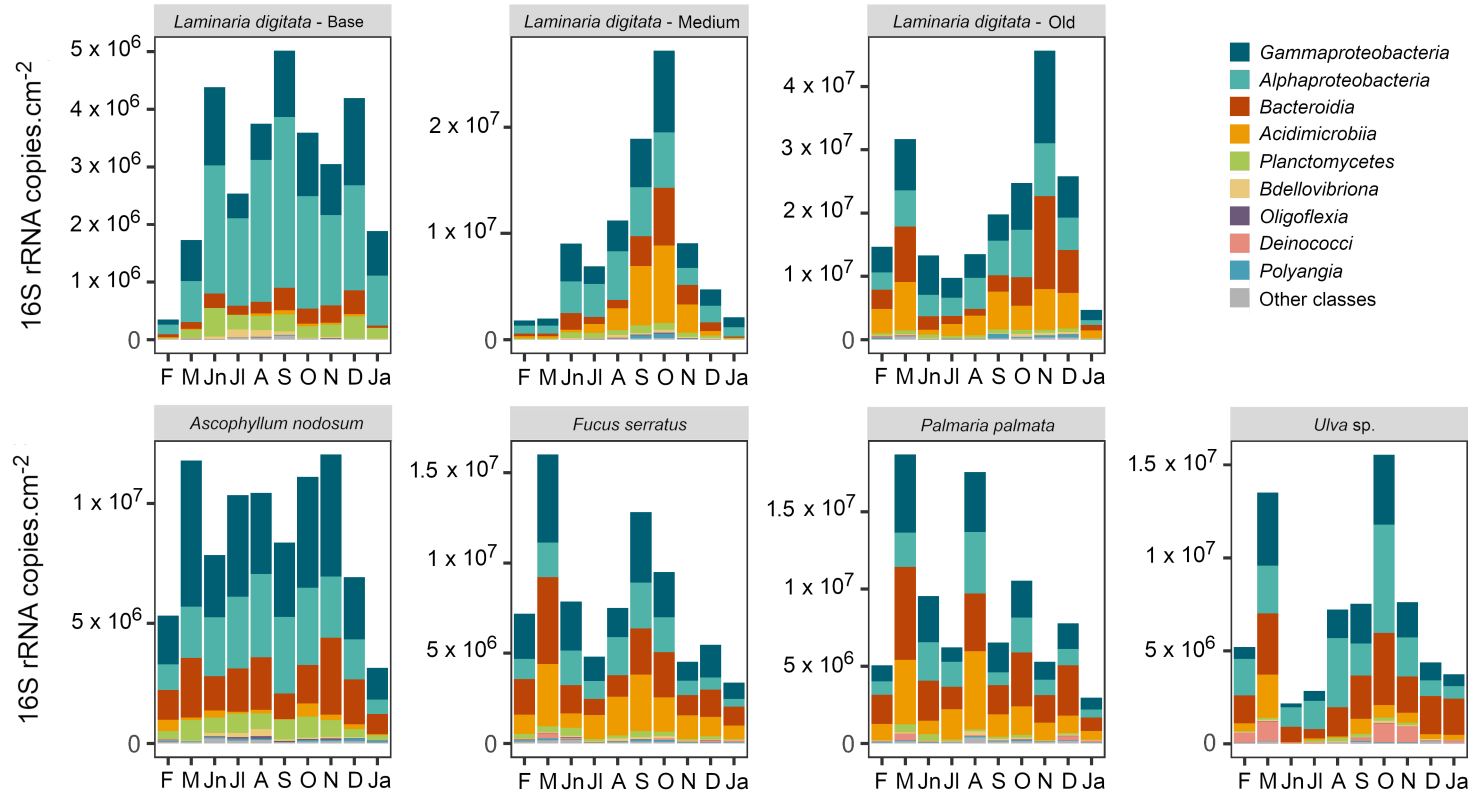

Supplement: Supplementary file 4 — Figure S4. Estimated abundance of the main bacterial classes. Only classes representing more than 3% (triplicate average) of the communities for at least one condition (i.e., at one time point for one algae) are shown. [file EMI4-17-e70077-s003.pdf]

Observed ASVs

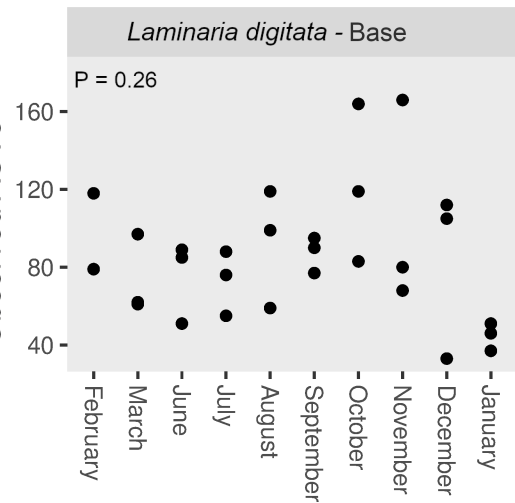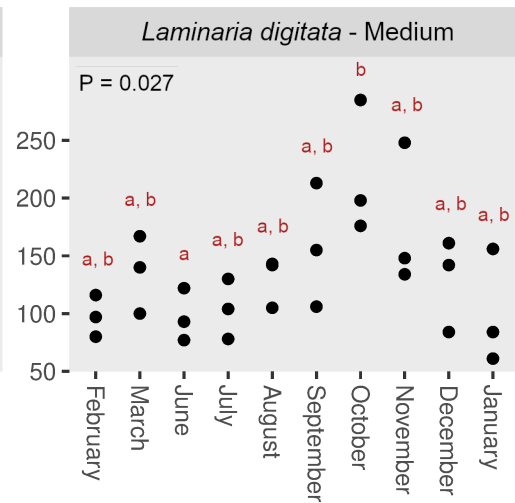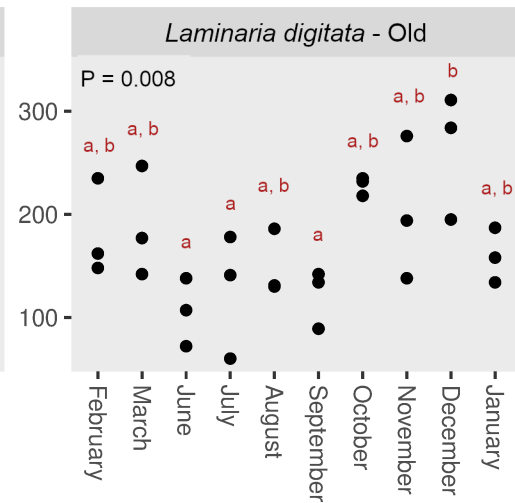

Observed ASVs

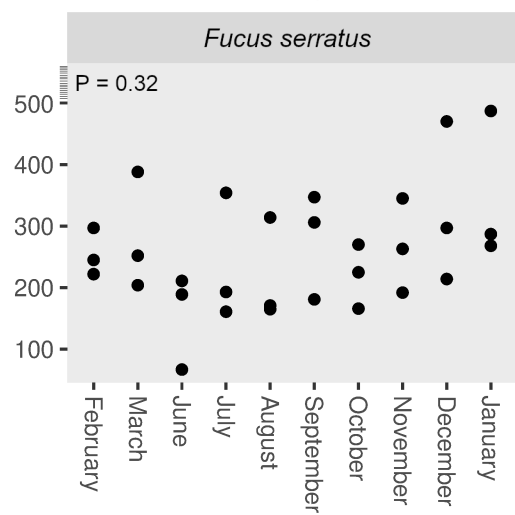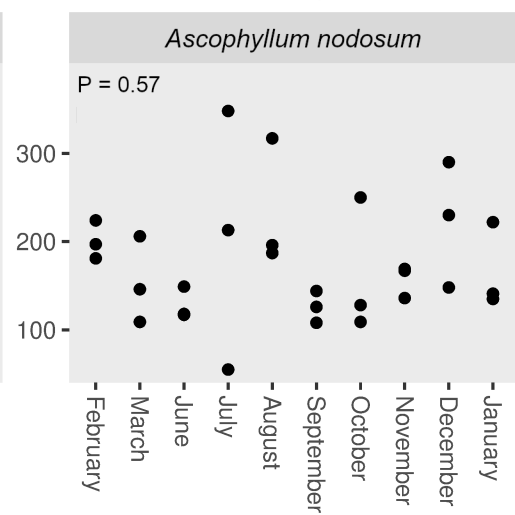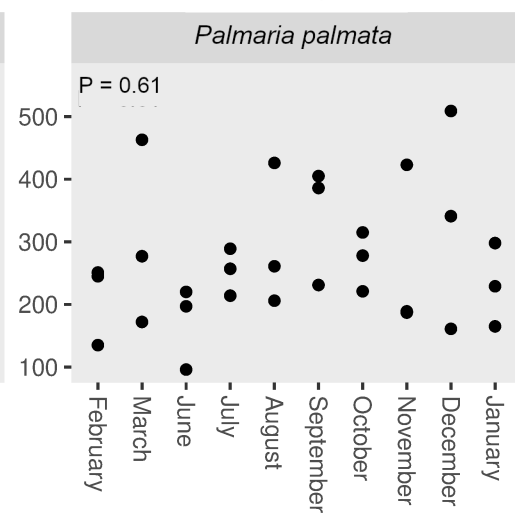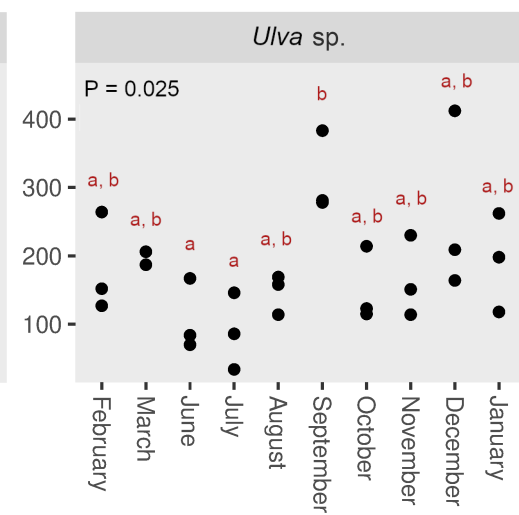

Supplement: Supplementary file 5 — Figure S5. Fluctuation of the number of observed ASVs. When significant ANOVA results were found (p < 0.05), a post hoc Tukey HSD test was calculated. Accordingly, different letters indicate significant differences between sampling months. [file EMI4-17-e70077-s010.pdf]
