# Supplementary material for: Year‐Round Quantification, Structure and Dynamics of Epibacterial Communities From Diverse Macroalgae Reveal a Persistent Core Microbiota and Strong Host Specificities
Source: Environ Microbiol Rep. 2025 Mar 12;17(2):e70077. doi: 10.1111/1758-2229.70077 (PMC11903338; doi:10.1111/1758-2229.70077)
Supplement: Supplementary file 8 — File S3. Differences in primer coverage. [file EMI4-17-e70077-s007.docx]

**Supplementary File S3: Differences in primer coverage**

The primers pairs respectively used for qPCR (926F / 1062R) and 16S rDNA metabarcoding (S-D-Bact-0341-b-S-17 / 799F_rc) have different predicted coverage. The table below summarizes the predicted coverage for several taxa as well as the observed number of ASVs and their relative abundance in the metabarcoding dataset.

| **Taxon** | **qPCR** predicted coverage^#^ | **Metabarcoding** predicted coverage^#^ | **Metabarcoding**  number of ASVs | **Metabarcoding**  average abundance (mean ± s.e.m, n=208) |
| --- | --- | --- | --- | --- |
| Eukaryota | 0.1% | 0.1% | 27 | 0.150 ± 0.037 % |
| Archaea | 0.5% | 58.3% | 0 | None detected |
| Bacteria | 94.1% | 80.6% | 10,243 | 99.6 ± 0.094 % |
| Mitochondria | 61.1% | 43.5% | 38 | 0.010 ± 0.003 % |
| Cyanobacteria (incl. chloroplasts) | 89.5% | 0.8% | 39 | 0.269 ± 0.087 % |
| # assessed on Silva Testprime against SSU r138.1 with only one mismatch allowed, analysis performed in December 2024 | | | | |

Both primer sets do not target Eukaryota. The qPCR primer set does not target Archaea, while the predicted coverage for the metabarcoding primer set is 58.3%. Yet, no archaeal ASV was detected in the metabarcoding dataset. Mitochondria have similar predicted coverage with both primer sets. The very low abundance of mitochondrial ASVs observed with metabarcoding (0.010% on average) shows that mitochondria were negligible in our samples. Therefore, removing the eukaryotic and mitochondrial ASVs prior to multiplying qPCR counts by metabarcoding relative abundance is not expected to affect the final estimate of bacterial 16S rRNA copy numbers. One important difference is the amplification of chloroplasts. The metabarcoding primer set was specifically designed to avoid plastid amplification in analyses of alga-associated bacterial communities (Thomas *et al.*, 2020). Therefore, the observed low abundance of cyanobacteria (including chloroplasts) in the metabarcoding dataset (0.269% on average) does not inform on the true abundance of chloroplasts in the analyzed samples. For this reason, we chose to also remove the 39 ASVs affiliated to cyanobacteria (including chloroplasts) from the metabarcoding dataset. By contrast, the qPCR primer set does target cyanobacteria (including chloroplasts). Multiplying qPCR total counts by metabarcoding relative abundance therefore likely over-estimates the individual absolute abundance of each ASV. However, the swab technique we used to sample epibacterial communities on macroalgae is known to avoid chloroplast contamination and retrieve low relative abundance of chloroplasts compared to extractions on whole algal tissues (Aires *et al.*, 2018), e.g. <2.5% on *Laminaria digitata* (Thomas *et al.*, 2020), <5% on *Fucus vesiculosus* (Stratil *et al.*, 2014) and <5% on Ulva sp. (Van Der Loos *et al.*, 2021). Hence, we estimate that the over-estimation bias is below 10% and does not change conclusions.

Aires, T., Muyzer, G., Serrão, E.A., and Engelen, A.H. (2018) Unraveling seaweeds bacteriomes. In *Protocols for Macroalgae Research*. Charrier, B., Wichard, T., and Reddy, C.R.K. (eds). CRC Press, pp. 95–113.

Stratil, S.B., Neulinger, S.C., Knecht, H., Friedrichs, A.K., and Wahl, M. (2014) Salinity affects compositional traits of epibacterial communities on the brown macroalga *Fucus vesiculosus*. *FEMS Microbiol Ecol* **88**: 272–279.

Thomas, F., Dittami, S.M., Brunet, M., Le Duff, N., Tanguy, G., Leblanc, C., and Gobet, A. (2020) Evaluation of a new primer combination to minimize plastid contamination in 16S rDNA metabarcoding analyses of alga‐associated bacterial communities. *Environ Microbiol Rep* **12**: 30–37.

Van Der Loos, L.M., D’hondt, S., Willems, A., and De Clerck, O. (2021) Characterizing algal microbiomes using long-read nanopore sequencing. *Algal Res* **59**: 102456.
